# Tackling the physiological barriers for successful mesenchymal stem cell transplantation into the central nervous system

**DOI:** 10.1186/scrt312

**Published:** 2013-08-22

**Authors:** Nathalie De Vocht, Jelle Praet, Kristien Reekmans, Debbie Le Blon, Chloé Hoornaert, Jasmijn Daans, Zwi Berneman, Annemie Van der Linden, Peter Ponsaerts

**Affiliations:** 1 Laboratory of Experimental Hematology, Vaccine and Infectious Disease Institute (Vaxinfectio), University of Antwerp, Campus Drie Eiken, Universiteitsplein 1, 2610, Antwerp (Wilrijk), Belgium; 2 BioImaging Laboratory, University of Antwerp, Campus Drie Eiken, Universiteitsplein 1, 2610, Antwerp (Wilrijk), Belgium

## Abstract

Over the past decade a lot of research has been performed towards the therapeutic use of mesenchymal stem cells (MSCs) in neurodegenerative and neuroinflammatory diseases. MSCs have shown to be beneficial in different preclinical studies of central nervous system (CNS) disorders due to their immunomodulatory properties and their capacity to secrete various growth factors. Nevertheless, most of the transplanted cells die within the first hours after transplantation and induce a neuroinflammatory response. In order to increase the efficacy of MSC transplantation, it is thus imperative to completely characterise the mechanisms mediating neuroinflammation and cell death following MSC transplantation into the CNS. Consequently, different components of these cell death- and neuroinflammation-inducing pathways can be targeted in an attempt to improve the therapeutic potential of MSCs for CNS disorders.

## Introduction

Stem cell transplantation is hoped to become a promising therapy for regeneration of injured central nervous system (CNS) tissue as numerous preclinical studies have already demonstrated improved functional outcome following stem cell transplantation [1-4]. Several potential working mechanisms have been proposed to explain their clinical benefit [5]; these are based on (i) immunomodulation, (ii) stimulation of endogenous neural stem cells and/or endogenous regeneration-inducing mechanisms by (genetically modified) cellular grafts, or (iii) direct cell replacement. More recently, insights into neuroinflammatory processes induced by stem cell transplantation might further explain possible contributions of stem cell transplantation neuroprotection and/or neurorestoration.

Despite the observed beneficial effects of stem cell grafting into the CNS, which might be attributed to one or more of the above described mechanisms, little is known about the actual mechanism responsible for the beneficial effects observed in different CNS diseases (stroke, Alzheimer’s disease, Parkinson’s disease, Huntington’s disease, spinal cord and traumatic brain injuries, and multiple sclerosis). Functional outcome following cell grafting demonstrates very diverse functional and pathological results, which might be due to differences in disease model, cell source and dose, application route and time window [6-11]. Whereas in the past researchers looked mainly at the functional benefits following stem cell transplantation, attention is now being paid to the fate (based on cell labelling with particles and/or reporter genes) and physiology (based on differentiation capacity and secretion potential) of the transplanted cells in order to reach a better understanding of the underlying mechanism. Looking into the cell fate, the survival of transplanted cells was poorly investigated and found to be very low [12-16]. While intravenous injection is the most feasible administration route, stem cell survival is very poor following intravenous injection as the cells become entrapped in filter organs such as liver, spleen and lung [17], where they die via apoptosis (within hours to a few days) [18]. Highest cell survival has been observed following cell transplantation into the CNS [19,20], despite the latter being shown to induce neuroinflammation at the site of injection. The latter has mainly been characterised by the recruitment of microglia and astrocytes in both healthy [21] and diseased CNS [9,22]. Alternatively, other research groups reported a decreased activation of microglia and astrocytes at lesion sites [6,12], as well as the production of anti-inflammatory cytokines leading to disease improvement [23-25] following mesenchymal stem cell (MSC) transplantation into the CNS. Given the low cell survival after transplantation, it might be possible that the cells themselves are not the key players in regeneration, but rather cell death-induced responses and subsequent (immunological) responses following cell transplantation. Therefore, it is imperative to thoroughly characterise cell survival and neuroinflammation following MSC transplantation, in order to gain better insights into the physiological responses leading to disease improvement and to find specific targets for therapeutic intervention.

Besides their successful therapeutic application based on their intrinsic properties, MSCs also form an interesting cell source for the secretion of growth factors and cytokines, supporting CNS disease improvement [26]. Adopting this approach, the beneficial effect is induced by the secreted factors, which can support endogenous neurogenesis and/or neuroprotection, and its success is highly dependent on stem cell survival and their potential to secrete growth factors. Low cell survival, due to hypoxia and serum deprivation, has already been reported following stem cell transplantation in myocardial infarction [27], and these are most likely also the causal factors for the low cell survival observed after stem cell transplantation into the CNS. Therefore, the use of trophic factor-producing MSCs for CNS disease treatment might hold promise for developing strategies to improve stem cell survival after transplantation, in order to obtain highly viable, growth factor-producing stem cells at the site of injury. In addition to establishing better cell survival, reducing the neuroinflammation is also of interest, as MSCs become surrounded by an astrocytic scar [20], probably induced by the microglial neuroinflammatory response. Such glial scarring may prevent the secreted growth factors from reaching their target, thus possibly reducing the therapeutic benefits.

Improved functional outcome after MSC transplantation for CNS disorders is attributed to neuroprotection, immunomodulation, or improved endogenous neurogenesis induced by the immunomodulatory signalling cascade, or by growth factors secreted by the transplanted stem cells. However, the therapeutic application of MSCs in CNS disorders is challenged by low cell survival following transplantation, as well as by the presence of neuroinflammatory responses. Therefore, a comprehensive characterisation of both neuroinflammation and the mechanisms underlying low cell survival following MSC transplantation is absolutely crucial for identifying possible targets that can be modulated to improve the therapeutic potential of MSC application.

### Neuroinflammation and neuroprotection

Neuroinflammation is a common characteristic of neurodegenerative and neuroinflammatory diseases, of which the presence of activated microglia is an important hallmark. It is imperative to recognise that microglia can be activated in different ways, resulting in distinct microglial functions during CNS disease. Below, we describe the general mechanisms behind the different types of microglia activation (classical versus alternative activation, and acquired deactivation), and discuss the neuroinflammation (as a type of microglia activation) observed after autologous transplantation of MSCs into the mouse brain.

### Classical activation of microglia

In most CNS disorders, microglia become activated in a similar fashion to when they respond to the presence of pathogens (classical activation), where certain ligands characteristic for CNS disease (for example, amyloid beta plaques, α-synuclein) act as pathogen-associated molecular patterns and are recognised via pathogen recognition receptors (PRRs) [28]. Toll-like receptors (TLRs) 1 to 9 are well-known PRRs expressed on microglial membranes [29], and different compounds associated with cell death or CNS diseases are known to activate microglia via TLR signalling [30]. Activation of TLRs can have two downstream effects: it leads to either nuclear factor (NF)-κB signalling via myeloid differentiation primary response gene 88 (MyD88) recruitment, or interferon regulatory factor (IRF)3 triggering upon TRIF-related adapter molecule-dependent TIR-domain-containing adapter-inducing interferon-β (TRIF) signalling [31]. Activation of either NF-κB or IRF3 leads to the nuclear translocation of both transcription factors, an event that induces the transcription of inflammatory cytokines (IL6, IL12, IL18, TNFα, IL1β) [29] and type I interferon [32], elicits the production of reactive oxygen and nitrogen species [33], and stimulates phagocytosis [34]. The production of reactive oxygen and nitrogen species leads to neuronal death, thereby further augmenting the ongoing neuroinflammation [35]. In addition, ATP is released into the extracellular environment by dying neurons, activating microglia and astrocytes. ATP triggers the purinergic receptor P2X_7_, present on microglial membranes [36], resulting in the production of neurotoxic cytokines (IL1β and TNFα) upon NF-κB signalling, consequently sustaining neuronal death [37]. Classic activation of microglia has been widely studied in CNS disorders, and can easily be stimulated *in vitro* via activation of TLR4 upon lipopolysaccharide treatment [38] or via activation of the interferon γ receptor with interferon γ. In contrast to lipopolysaccharide, which activates NF-κB through TLR4, interferon γ triggers Janus kinase signal transducer and activator of transcription (Jak/STAT) signalling to induce the production of pro-inflammatory cytokines [39]. In general, this type of classical microglia activation is considered detrimental for the disease, although a few cases have been reported in which classically activated microglia can be supportive for remyelination [40] and microglial phagocytosis has a beneficial effect on Alzheimer’s disease [41].

### Alternative activation of microglia

In addition to classical activation of microglia, which generally results in neuroinflammation, microglia can acquire a more neuroprotective, alternatively activated phenotype. Alternative activation can be obtained via cytokine (IL4 and IL13) signalling through their common IL4Rα receptor followed by Jak/STAT signalling and activation of the transcription factor STAT6 [42]. Subsequent nuclear translocation of STAT6 leads to the transcription of anti-inflammatory genes and the transcription factor peroxisome proliferator-activated receptor (PPAR)γ, which has an inhibitory effect on the expression of pro-inflammatory genes upon classical microglia activation [43]. Furthermore, the production of reactive nitrogen and oxygen species is reduced, whereas the release of anti-inflammatory cytokines (IL10, transforming growth factor (TGF)β) is promoted, resulting in neuroprotection. Although the spontaneous alternative activation (M2) of microglia has not been well-described *in vivo* for CNS disorders and is largely overruled by a neurotoxic M1 response [44], it is believed that the presence of M2 microglia is supportive in CNS disease and is able to suppress the neurotoxic effects of classically activated microglia.

### Acquired deactivation

Following classical and alternative activation of microglia, the quiescent phenotype of microglia can be restored through a process of microglial deactivation [45,46]. This acquired microglia deactivation has been studied thoroughly in tumour models in which the tumours are a source of anti-inflammatory cytokines (IL4, IL6, IL10 and TGFβ) [47] that induce microglia deactivation through signalling via their receptors (IL4R, IL6R, IL10R and TGFβR), leading to the suppression of immune responses against the tumour [48]. IL4R ligand binding triggers the Jak/STAT signalling cascade and subsequent transcription of PPARγ, as discussed previously (section on alternative microglia activation). In addition, IL10 and TGFβ, cytokines produced upon alternative activation of microglia, give rise to a quiescent microglia phenotype through signalling via their respective receptors, IL10R and TGFβR [49]. IL10, TGFβ and corticosteroids are also upregulated upon phagocytosis of apoptotic cells [50]. Therefore, both classical and alternative activation of microglia, each characterised by distinct cell functions and cytokine production, might induce microglia deactivation [49] and subsequently a reduction of neuroinflammation.

### Neuroinflammation following mesenchymal stem cell transplantation

**Figure 1:**
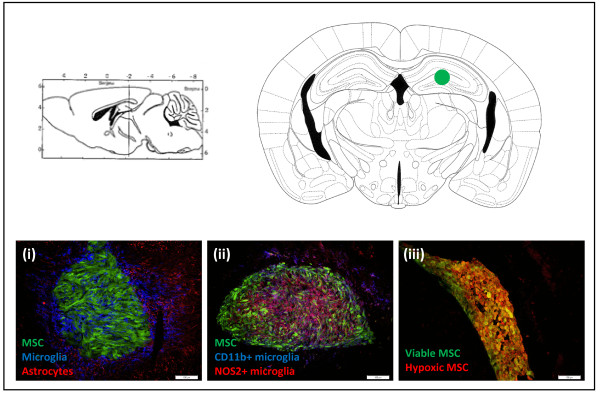
**Characterisation of responses following mesenchymal stem cell transplantation.** The upper panel of Figure 1 demonstrates a transveral and coronal brain slice at the site where mesenchymal stem cells (MSCs) were transplanted (green dot). The bottom row of Figure 1 demonstrates immunofluorescent pictures of cryosections from FVB mice transplanted with 2 × 10^5^ enhanced green fluorescent protein (eGFP)- and luciferase-expressing MSCs into the striatum. **(i)** Transplanted MSCs are recognised by the brain’s immune system, demonstrating Iba1+ microglia (blue) invading and activated GFAP + astrocytes (red) surrounding the eGFP-expressing MSC implant (green) at week 1 post-implantation. **(ii)** A proportion of the Iba1+ microglia are classically activated and demonstrate CD11b (blue) and NOS2 (red) expression, corresponding to a neuroinflammatory microglial phenotype. **(iii)** At 6 h post-transplantation a lot of hypoxic (red) cells are found within the eGFP-positive (green) MSC transplant, leading to death of about half of the transplanted cells. The representative pictures were taken at a magnification of 20 × .

Although MSC transplantation into the CNS has been ascribed beneficial effects in numerous preclinical studies of neurodegenerative and neuroinflammatory disorders [1], a minority of studies report stem cell survival and its influence on neighbouring tissue upon transplantation into the brain. However, when a follow-up of stem cell behaviour after transplantation was performed, the survival of MSCs was found to be very low (between 0 and 30%) and was highly dependent on the injection route/site (intravenous, intracerebral or intrathecal) and cell source (auto-, allo- or xenogeneic) [2-6]. Our research group previously demonstrated the ability of autologous MSCs to survive after transplantation into the CNS [20,51], whereas no cell survival was observed after transplantation into the muscle [51] or following intravenous administration [18]. Nevertheless, cell survival was only observed for autologous, but not for allogeneic or xenogeneic stem cell grafts, which are rejected via a T cell-independent mechanism [33]. By accurately quantifying the survival of autologous CNS-implanted MSCs, we demonstrated that only a fraction of the initially implanted cells are able to survive in both healthy [21] and diseased [52] CNS. In addition to the low cell survival after transplantation, an increased neuroinflammatory response, characterised by microgliosis and astrogliosis, was observed after MSC transplantation in healthy CNS [21,53] (Figure 1i). We noted that microglia have the capacity to recognise the presence of autologous MSCs and become activated in a classical way, demonstrated by the expression of inducible nitrogen oxide synthase (Figure 1ii). Although MSCs have been shown to have neuroprotective features when administered in models of CNS disease [6,12,54-57], they are able to induce pro-inflammatory responses at the site of injection in healthy [21] or diseased animals [52]. Consequently, this inflammatory cascade induced by stem cell application needs to be considered and possibly even modified in the event of stem cell transplantation, in order to prevent possible neurotoxic effects and optimise the therapeutic potential of stem cells.

Below, we describe different strategies to modify neuroinflammation, which may be adopted to eliminate undesirable immune responses induced by stem cell applications.

### Strategies for the modulation of neuroinflammation

In general, brain injury results in microglia activation (either classical or alternative), which can be beneficial or detrimental for different types of CNS diseases [58]. Directed modification of the microglial activation status could positively influence the disease course, thereby offering great potential as a therapeutic strategy. Moreover, this approach also holds promise for altering the immunological responses observed after stem cell transplantations, with the aim of decreasing neurodestructive immune responses at the site of injection. In order to use the adequate approach (stimulation or inhibition of neuroinflammation and/or neuroprotection), it is imperative to start by defining the activation status of microglia and their function in CNS disease or following stem cell application. Exact specification of the type of microglia activation may prove challenging, as it is highly dependent on the type of stimulated PRR, the location of microglia in the CNS and the time point during the disease course. Applying this concept to an example such as stroke, cytotoxic microglia are observed in the striatum, whereas subventricular zone microglia promote neurogenesis [59]. Furthermore, mouse models for amyotrophic lateral sclerosis exhibit neuroprotective microglia at disease onset, which are gradually transformed into cells with a more neurotoxic phenotype at the end of the disease [60]. Therefore, therapeutic intervention at the right place and time is vital to achieve success. Strategies can either focus on the inhibition of microglia activation by maintaining tissue homeostasis, or the modification of the activation status (classical or alternative activation or acquired deactivation) of microglia in order to attain neuroprotection. Based on the complex microglial activation signalling, modulation of neuroinflammation can be exerted at different levels of the signalling pathway: PPARs, TLRs, NF-κB signalling, Jak/STAT signalling, ATP signalling via purinoreceptors. Within the scope of this review we will, however, only focus on neuro-immunomodulating targets that hold great potential to be influenced via the application of (un)modified MSCs.

### Mesenchymal stem cells for the inhibition of microglia activation

MSCs are generally believed to induce immunosuppression via the production of different immunomodulatory factors (for example, nitric oxide, indoleamine 2,3-dioxygenase, TGFβ) that can inhibit T-cell proliferation, induce tolerogenic dendritic cells or prevent/polarise T-cell activation [61]. Although the immunomodulatory potential of MSCs and its relation to cells from the peripheral immune system has well been described, so far little is known about the role of MSCs in neuroinflammation. Recently, it became clear that MSCs also have the potential to influence neuroinflammation, both on the level of inhibiting microglia activation as well as modulation of microglia activation. Within the scope of this review we will only describe the effect of cytokines produced by MSCs that have an effect on neuroinflammation mediated by microglia.

Under steady state conditions, microglia display a ramified morphology and exert surveillance of the brain in order to accurately respond to danger signals [62]. Different mechanisms involving microglia-neuron signalling are able to maintain these steady state conditions, while the disruption of this cross-talk results in microglia activation [49].

### CX3CR1-CX3CL1

Fractalkine (CX3CL1) is known to be an important compound in microglia-neuron interaction. CX3CL1 exists both as a membrane-bound and as a secreted protein [63]. The secreted form functions as a chemoattractant for inflammatory cells while the membrane-bound form induces the adhesion of cells expressing the fractalkine receptor (CX3CR1) [64]. The binding of CX3CL1, expressed on neurons, to its receptor suppresses microglia activation [49]. Following CNS injury or disease, CX3CR1-CX3CL1 homeostasis is frequently disturbed, leading to either increased neuroprotection or disease worsening.

While neurons secrete higher levels of CX3CL1 upon CNS injury [65], leading to neuroprotection via a downregulation of pro-inflammatory cytokine production (nitric oxide, IL6, TNFα) by activated microglia [66], decreased neuroinflammation has been described for Alzheimer’s disease [67,68] and stroke [69] in CX3CR1^−/−^ mice. An explanation for the latter can be found in the chemotactic properties of fractalkine resulting in the absence of migration of activated microglia towards CNS lesions and subsequent neuroprotection upon CX3CR1 silencing. Alternatively, deletion of CX3CR1 leads to enhanced phagocytosis of amyloid beta plaques by microglia in Alzheimer’s disease, requiring the microglia to be present at the lesion sites [70].

On the other hand, the complete depletion of CX3CR1 has been demonstrated to worsen Parkinson’s disease, amyotrophic lateral sclerosis [71], experimental autoimmune encephalomyelitis (EAE) [72] and Alzheimer’s disease [73]. In addition, CX3CR1 deficiency leads to severe cognitive deficits in healthy animals [74]. As demonstrated above, the chemotactic properties of fractalkine might play an important role in microglia recruitment towards CNS injured sites. Complete elimination of activated microglia may prove to be detrimental as for some CNS diseases the presence of (neuroprotective) microglia is absolutely necessary in order to obtain a clinical benefit [58].

Since the above-mentioned studies show contradictory results about CX3CR1 signalling in Alzheimer’s disease, and describe a dual role for CX3CR1-CX3CL1 signalling in neuroinflammation, attention needs to be paid while modifying CX3CR1 or CX3CL1 expression in order to improve disease outcome. Whereas increased recruitment of activated microglia towards a site of CX3CL1 expression can be desirable in some situations (by the intracerebral injection of fractalkine in rodent brain [75]), a complete lack of CX3CL1 signalling may be preferred in other instances (using a CX3CR1 antagonist, which inhibits the CX3CL1-induced chemotaxis [76]).

For future applications, MSCs hold great potential as a strategy to modulate neuroinflammation via CX3CR1-CX3CL1 signalling, as *in vitro* experiments demonstrated that they are able to produce CX3CL1, thereby directing microglia towards a more neuroprotective phenotype [56,77]. Neuroprotection induced by CX3CL1 production by MSCs was mediated by the downregulation of neuroinflammatory cytokine production (IL1β and TNFα) and upregulation of TREM2, CX3CR1 and CD200R expression on microglia. In addition, *in vivo* MSC transplantation has been shown to reduce stroke-related neuroinflammation via the downregulation of the expression of the pro-inflammatory inducible nitrogen oxide synthase gene and decreased accumulation of microglia and astrocytes at the site of ischaemia, possibly induced via the production of fractalkine [56].

In conclusion, for CX3CR1 to be used as a target to influence neuroinflammation, the approach will need to be adapted to each type of CNS disease. In this regard, it is very important to determine if the presence of deactivated/alternatively activated microglia or complete absence of microglia will be supportive for obtaining neuroprotection. Therefore, treatment strategies need to focus on microglia deactivation, in which case the re-establishment of CX3CR1-CX3CL1 homeostasis is pursued, or on microglia elimination, by counteracting the chemotactic properties of CX3CL1 [76].

### CD200R-CD200

Microglia activation can also be suppressed upon interaction of various other microglia receptors (CD172, CD200 receptor (CD200R) and CD45) with markers expressed on neurons (CD47, CD200 and CD22) [78]. So far, a substantial amount of research has been performed towards elucidating the role of CD200-CD200R signalling in neuroinflammation, which generally results in increased microglia activation and neuroinflammation upon decreased CD200-CD200R interaction.

Increased numbers of hyperactive microglia were observed in CD200^−/−^ mice [79] and in aged animals exhibiting a lower CD200 expression [80]. Moreover, CD200 deficiency leads to an earlier EAE onset [79] but can nevertheless be helpful in the clearance of certain parasites via increased inflammation [81]. In contrast, upregulation of CD200 expression on neurons in *wld* mutant mice results in a decreased susceptibility to EAE [82], and CD200 upregulation upon kainic acid-induced neurodegeneration might play a role in alternative microglia activation [83].

In general, physiological CD200-CD200R signalling should be either maintained or stimulated, via the increased expression of CD200 and CD200R, to promote neuroprotection. For example, IL4 treatment promotes CD200 expression on microglia, suppressing their activation [80,84,85], thereby creating novel possibilities for neuroprotection in Alzheimer’s disease [86]. Stimulation of CD200-CD200R signalling via an agonistic anti-CD200R antibody can be beneficial in different autoimmune diseases, particularly for the resolution of experimental autoimmune uveoretinitis [87]. Additionally, microglia on their own are able to express CD200 upon excitotoxic lesioning, which was not observed in healthy conditions. Therefore, microglia themselves are able to contribute to neuroprotection and tissue homeostasis through the expression of both CD200 and CD200R [85]. Alternatively, increased numbers of activated microglia as well as an upregulation of the production of pro-inflammatory cytokines was observed upon blocking of CD200-CD200R signalling with a CD200R blocking antibody [88].

*In vitro* studies have demonstrated that MSCs are able to express CD200, and consequently can induce a CD200 expression level-dependent reduction in TNFα production by macrophages, resulting in increased neuroprotection [89]. As TNFα is a well-known cytokine produced by classically activated macrophages and microglia that contributes to neuroinflammation, downregulation of its production is a highly recommended strategy to modulate neuroinflammation. Despite the evidence of CD200 as a promising target molecule in immune-modulating strategies, to date there are no reports on the modulation of CD200-CD200R signalling upon MSC transplantation into the CNS.

### Mesenchymal stem cells for the modification of microglia activation

While the above-described approaches aim at preventing microglia activation, neuroprotection can also be stimulated via the reorientation of the microglia neurotoxic activation state (classical activation) towards a more neuroprotective activation state (alternative activation). Despite the fact that neuroinflammation occurs following MSC transplantation in healthy [21] and diseased [52] brain, many researchers ascribe neuroprotective effects to MSCs [90]. *In vitro*, MSCs have been shown to reduce the TNFα and nitric oxide production of lipopolysaccharide-stimulated microglia [91,92] and to suppress the production of pro-inflammatory cytokines in co-culture with astrocytes [93]. Therefore, the application of MSCs on their own might hold great potential as a strategy to modify neuroinflammation *in vivo*. Following this approach, neuroprotection can be induced via alternative activation of microglia following MSC transplantation in spinal cord injury [25], multiple sclerosis and Alzheimer’s disease [23,24,94]. In addition, MSCs can be used as carriers to secrete IL4, changing the microglial activation state towards a more neuroprotective phenotype in EAE [95]. The latter approach might also be an ideal immunomodulating strategy to treat other CNS disorders.

### Mesenchymal stem cell applications for trophic factor delivery

Previously, the CNS was believed to be immune privileged due to the presence of the blood brain barrier and the absence of a lymphatic system [96,97], suggesting the possible long-term survival of CNS-transplanted stem cells. However, it recently became widely accepted that specific immune responses, orchestrated by microglia and astrocytes, are able to fight foreign tissue (such as stem cells) in the brain [98,99]. Consequently, research reoriented towards the follow-up of stem cell survival and fate after transplantation into the CNS, including stem cell labelling with particles or dyes and/or reporter genes. Despite the fact that reporter gene labelling enables us to follow up the cell fate very accurately, only a minor number of studies included the determination of cell survival in a quantitative manner [12-16], demonstrating low cell survival (0 to 30%) upon transplantation. Although the invasion of the brain’s innate immune cells, starting on day 3 following autologous transplantation of MSCs into the CNS, represents a possible trigger for cell death [21], most of the transplanted cells die during the first few hours after transplantation, at a time when immune cells are not yet involved. As the transplanted MSCs become hypoxic within the first 24 hours following transplantation (Figure 1iii), it is most likely that hypoxia and serum deprivation are the major contributors to early cell death following transplantation. Although low cell survival due to hypoxia and serum deprivation has already been widely investigated for myocardial infarction [100], this observed low cell survival following autologous MSC transplantation into the CNS has not yet been thoroughly explored. In culture conditions, cells are provided with oxygen and nutrients in order to allow them to grow and proliferate. However, when cultured stem cells are used for transplantation experiments, they are removed from their oxygen- and nutrient-rich environment and placed in an injured (hypoxic) tissue environment without adequate oxygen and nutrient supply, leading to cell death via apoptosis. The success of stem cells as carriers for growth factors in therapeutic approaches for CNS diseases relies on their ability to survive and engraft in the surrounding tissue, in order to sustain growth factor production. The production of brain-derived neurotrophic factor (BDNF), glial cell line-derived neurotrophic factor (GDNF), vascular endothelial growth factor (VEGF) and/or neurotrophin 3 (NT3) can be very supportive in stroke [101-103], Huntington’s disease [104], Parkinson’s disease [26,105,106], spinal cord injury [107,108], and traumatic brain injury [109,110]. Therefore, it is important to obtain cells with high survivability following transplantation in order to obtain high expression levels of the supportive growth factors *in vivo*. Further research on the roles of hypoxia and serum deprivation in cell death after transplantation into the CNS will be necessary in order to develop adequate strategies to circumvent the problem of low cell survival. Below, we give an overview of strategies that can be adopted to face these problems of hypoxia and serum deprivation, thereby increasing the therapeutic potential of stem cell applications.

### Strategies for the improvement of cell survival

#### How to deal with hypoxia?

Hypoxic preconditioning can be applied to MSCs in culture prior to transplantation, in order to adapt the cells to the environmental conditions they will need to handle after transplantation. Cell culture in hypoxic conditions (1 to 3% oxygen) will increase their expression of pro-survival and pro-angiogenic genes [111], which can be beneficial for the survival of the cells as they are better adapted to reside in a more hypoxic environment, for example, brain tissue. The upregulation of hypoxia-inducible factor (HIF)-1α is a general consequence of hypoxic culture conditions and leads to enhanced expression of stromal-derived factor (SDF)-1α [112] and CXCR4 [113]. This attributes a special role to the SDF-1α/CXCR4 axis in cell survival [114,115] via the inhibition of caspase 3 activity through increased Akt phosphorylation (Table 1). Therefore, stimulation of the SDF-1α/CXCR4 signalling axis via hypoxic preconditioning or genetic modification of MSCs in order to induce secretion of SDF-1α/CXCR4 has been shown to enhance MSC survival [116,117]. So far, it has already been described that transplanted MSCs are able to survive better upon hypoxic preconditioning in mouse models of myocardial infarction [118,119], stroke [120,121] or renal ischaemia [116]. In addition to HIF-1α, other pro-angiogenic factors, such as erythropoietin and VEGF, also become upregulated upon hypoxic cell preconditioning. Consequently, additional administration of one of these factors can be applied as an alternative strategy to expensive hypoxic preconditioning. While erythropoietin already demonstrated a beneficial effect on the survival of different kinds of progenitor cells after transplantation [122-124], HIF-1α-transduced MSCs significantly improved myocardial infarction [125] and VEGF-overexpressing neural stem cells demonstrated higher cell survival in an injured spinal cord [126]. Attention needs to be paid to additional genetic or phenotypic alterations that might be introduced accidentally upon modification of MSCs in order to produce hypoxia-resistant cells. Therefore, research efforts should be focused on the development of oxygen-generating biomaterials [127] or oxygen-permeable chips [128] that enable an increased oxygen supply to the graft without further adaptations to the graft.

**Table 1:** Overview of different approaches for mesenchymal stem cell modulation in order to increase cell survival

**General effect**	**Strategy**	**Reference**
Upregulation of different pro-survival and pro-angiogenic genes (HIF1α, SDF1α/CXCR4, EPO, VEGF, BDNF, GDNF)	Hypoxic preconditioning	[112,113]
	Addition of factors to the culture medium	[122,124,133]
	Genetic modification to induce gene overexpression	[125,126,129-132]
Physical protection of MSCs against hypoxia	Oxygen supply via oxygen generating scaffolds and biomaterials	[127,128]
Stimulation of PI3K/Akt pathway to prevent apoptosis	Treatment with chemokines (for example, SDF1α)	[138]
	Knockout of TLR4	[137]
	Overexpression of genes involved in apoptosis	[139]
Downregulation of caspase 3 activity to prevent apoptosis	Treatment with compounds (carvedilol, salvianolic acid) that block the activity	[140,141]
Decreased apoptosis	Down- or upregulation of microRNA	[142]

BDNF, brain-derived neurotrophic factor; EPO, erythropoietin; GDNF, glial cell line-derived neurotrophic factor; HIF, hypoxia-inducible factor; MSC, mesenchymal stem cell; PI3K, phosphoinositide 3-kinase; SDF, stromal-derived factor; TLR, Toll-like receptor; VEGF, vascular endothelial growth factor.

### How to deal with serum deprivation?

*In vitro*, the survival of certain cell types (for example, neural stem cells) is highly dependent on the availability of growth factors in the culture medium. Upon transplantation, however, these cells are deprived of these compounds necessary for survival and undergo cell death. Genetic modification of MSCs [129-132] or cell treatment with minocycline [133] in order to overexpress some of these essential growth factors (GDNF, BDNF, VEGF, survivin; Table 1) have already been applied in cell transplantation experiments for CNS disorders and has been shown to induce functional improvement of the disease. However, only few studies [134,135] reported increased survival of the transplanted cells upon growth factor treatment. Therefore, further research is imperative in order to understand how serum deprivation can be tackled with the aim of favouring cell survival.

The general consequence of hypoxia and serum deprivation is cell death mediated via apoptosis. Therefore, aside from manipulating the oxygen and serum supplies themselves, it is also possible to interfere with apoptosis via the stimulation of the phosphoinositide 3-kinase (PI3K)/Akt pathway. In this context, it is known that TLR4 contributes to hypoxia-mediated apoptosis [136], and that TLR4 knockout in MSCs leads to improved survival after hypoxic treatment [137]. Additionally, inhibition of hypoxia- and serum deprivation-induced apoptosis via activation of PI3K/Akt can also be obtained via cell treatment with chemokines [138] or by means of overexpressing (or downregulating) genes regulating the apoptotic process [139] (Table 1). Inhibition of caspase 3 activity via cell treatment with carvedilol [140] or salvianolic acid [141] leads to decreased apoptosis and subsequent improved cell survival (Table 1). Although the underlying mechanism is still unknown, an important role has been ascribed to the up- or downregulation of microRNAs in preventing apoptosis [142] (Table 1).

To summarise, many targets can be used in order to make MSCs more tolerant to hypoxic conditions, including those that (i) have a direct effect on the PI3K/Akt signalling pathway, (ii) modulate caspase 3 activity, (iii) enhance cell survival via different growth promoting factors (indirect and direct cell treatment or genetic modification) or (iv) act via the modification of microRNA expression.

Although some of the above-described strategies have the potential to ameliorate cell survival upon transplantation, research on cell survival after transplantation into the CNS and subsequent modification strategies is still in its infancy. A more profound characterisation of the cell death-inducing mechanisms will help us to understand how to appropriately prevent cell death following transplantation and obtain efficient carriers to deliver supportive growth factors.

### Drawbacks of mesenchymal stem cell modification to improve their potential to treat central nervous system disorders

While many of the above described strategies hold promise to ameliorate the capacities of MSCs to prevent neuroinflammation or hypoxia-mediated cell death upon grafting in the CNS, we need to take into account that several of these approaches require genetic modification of MSCs. One of the major concerns is that these genetic alterations might alter general properties of the MSCs, possibly leading to tumour formation following transplantation [143]. On the other hand, we also should assess the impact of different *in vitro* manipulations of stem cells, in order to increase cytokine and growth factor (BDNF, VEGF) secretion, on the direct surrounding of the transplanted stem cells *in vivo*. The secreted growth factors might on their own lead to aberrant tissue formation, possibly leading to tumour formation arising from endogenous cells. Profound long-term preclinical evaluation after transplantation of modified MSCs is therefore absolutely necessary in order to claim a clinical cell product is safe.

## Conclusion

Over the past decade there has been a growing interest in the use of MSCs to treat different neurodegenerative and neuroinflammatory diseases. Although various research groups ascribe neuroprotective and immunomodulatory properties to MSCs, their survival upon transplantation remains very poor. Moreover, conflicting data regarding their neuroprotective effect in CNS disorders have emerged in which MSCs have been described to induce a pro-inflammatory immune response, characterised by the presence of activated microglia and astrocytes, at implantation sites.

In order to optimise the therapeutic potential of MSC applications for neurodegenerative and inflammatory diseases, it thus becomes very important to completely characterise (and possibly modulate) neuroinflammation in CNS diseases as well as following MSC transplantation, and improve cell survival upon transplantation. As it is known that microglia are the major key players in neuroinflammation in many CNS disorders as well as following MSC transplantation, even without the contribution of T cells and other peripheral immune cells, new insights into microglia activation and the underlying mechanisms of neuroinflammation will be helpful in understanding the role of the brain’s innate immune system in CNS disease. Consequently, modulation of neuroinflammation can be employed, whether or not through the use of MSC transplantation, in order to ameliorate different neuroinflammatory diseases or to reduce the neurotoxic effect induced upon MSC transplantation itself. Finally, research on hypoxia and serum deprivation following stem cell transplantation into the CNS will broaden our knowledge on efficient approaches for increasing cell survival and optimising therapeutic stem cell-based implementations.

While applying different approaches in order to ameliorate stem cell-based beneficial effects on CNS disorders, we need to take into account the differences between animal models and human diseases, as well as potential drawbacks of genetic modification of transplanted stem cells before application in clinical trials.

## Abbreviations

BDNF: Brain-derived neurotrophic factor; CD-R: Cluster of differentiation receptor; CNS: Central nervous system; CX3CL1: CX3C chemokine ligand 1 (fractalkine); CX3CR1: CX3C chemokine receptor 1 (fractalkine receptor); CXCR4: C-X-C chemokine receptor type 4; EAE: Experimental autoimmune encephalomyelitis; GDNF: Glial cell line-derived neurotrophic factor; HIF: Hypoxia-inducible factor; IL: Interleukin; IL-R: Interleukin receptor; IRF: Interferon regulatory factor; Jak/STAT: Janus kinase signal transducer and activator of transcription; MSC: Mesenchymal stem cell; NF: Nuclear factor; PI3K: Phosphoinositide 3-kinase; PPAR: Peroxisome proliferator-activated receptor; PRR: Pathogen recognition receptor; SDF: Stromal-derived factor; TGF: Transforming growth factor; TGFβR: Transforming growth factor β receptor; TLR: Toll-like receptor; TNF: Tumour necrosis factor; VEGF: Vascular endothelial growth factor.

## Competing interests

The authors declare that they have no competing interests.
